# Keyword index to Volume 81

**DOI:** 10.1038/sj.bjc.6690865

**Published:** 1999-12


					Acetaminophen 542
N-Acetyltransferase 1 537
N-Acetyltransferase 2 537
Actin 393
Acute lymphoblastic leukaemia 175
Acute myeloid leukaemia 900
Adenocarcinoma 159
Adenoma 232
Adenovirus vectors 277
Adolescence 167
Adrenal cortex 684
Adrenocortical tumours 300
Advanced non-small-cell lung cancer 846
Adverse effects 336
Aetiology 898, 907
Affective disorders 907
Age-period-cohort analysis 152
Age-period-cohort models 159
Agreements 87
ALA cream 13
ALA lotion 13
ALA-PDT 13
Alcohol abuse 907
Alkaline sphingomyelinase 232
Allelic imbalance 1111
5-Aminolaevulinic acid 13
Amplification 1174, 1410
Analgesics 542
Anatomic sites 528
Androgens 28
Androgens, synthesis of 622
Androgen ablation 242
Androgen receptors 242, 672
Angiogenesis 54, 225, 354, 647, 1088, 1335,
1363, 1398
Angiosarcomas 532
Angiostatin 1269
Animal tumour models 638
Anthracyclines 24
Anthropometry 1257
Anti-angiogenesis 1269
Anti-vascular shutdown 1318
Antibodies 769
Antimicrobial peptides 393
Antisense interleukin-2 oligonucleotides 822
Antisense-EGFR 994
AP-2 133
APC protein 850
Apoptosis 277, 294, 387, 423, 520, 592, 747,
822, 952, 981, 1274, 1285, 1294
Arsenic trioxide 796
Asia, south east 893
Aspirin 294, 542
Astrocytomas 835
Atomic bomb survivors 1248
ATPase 269
Autoantibodies 702
Autocrine regulation 1335
Automatic quantification 1363
Azolyl steroids 622
Background transcription 870
BAG-1 1042
bcl-2 252
Bcl-2 387, 860, 1042
Bcl-x(L) 387
Benzamides 981
Berberine 416
Bi-specific monoclonal antibodies 431
Bicalutamide 242
Biological factors 925
Biomarkers 1222, 1238
Bioreductive drugs 1127
Biotinylated anti-CD3 Fab fragments 431
Bisdioxopiperazine 800
BK virus 898
Bladder cancer 537, 832
B-lymphoma cells, human 43
Body mass indices 890
Bombesin receptors 966
Bone marrow 1213
Brain tumours 1371
BRCA1 179
BRCA1 503
BRCA2 179
BRCA2 503
Breast cancer 225, 287, 316, 342, 387, 440,
532, 592, 690, 702, 850, 912, 1002, 1042,
1142, 1248, 1304, 1385
Breast cancer cells 252
Breast neoplasms 167, 490
Breast size 1257
Bromodeoxyuridine 404
Buthionine sulphoximine 796
C1311 367
CA19-9 769
CA 125 662
Cachexia 80
Calcium 219
cAMP analogues 1134
Cancer chemotherapy 69
Cancer invasion 721
Cancer risks 672
Cancer screening 554
Cancer testis antigens 1080
Cancer therapy 966
Cancer xenografts, human 203
Carboplatin 336
Carcinomas 592, 1059, 1363
Carcinomas, human 822
Cardiotoxicity 24
Carotenoids 1234
Case-control studies 62, 559, 890
Caspases 294, 592
-Catenin 1392
Cathepsin-B 510
Cathepsin-D 1385
CD34 808
Cell adhesion 381
Cell cultures 814
Cell cycles 808, 1285
Cell cycle checkpoints 959
Cell cycle regulators 696
Cell lines 814
Cell proliferation 712
Cell transformation 790
Cell-cell interaction 1142
c-erbB-2 1385
c-erbB-2 oncogenes 790
Cervical cancer 95, 108, 159, 554, 989
Cervical dysplasia 1234
Cervical scrapings 114
CGH 300, 1328, 1410
CHART 1196
Chemotherapy 75, 316, 336, 469, 484, 520,
835, 860, 1022, 1031, 1213, 1280
Chemotherapy, high-dose sequential 449
Chemotherapy responses 1017
Childhood 900
Childhood cancer 549
Childhood leukaemia 144
Children 175, 336, 835
Chromogranin A 667
Chromosomal aberrations 1328
Chromosome 3 684
Chromosome 9 684
Chromosome 17q 1402
Chromosome 22 1150
Chromosome X 684
Cigarette smoking 537
Cimetidine 1356
Circulating cancer cells 1066
Circulating epithelial cells 832
Circulating tumour cells 1165
Cisplatin 28, 95, 469, 1294
Clinical outcomes 696, 1174
Clinical trials 1356
Clonogenicity 756
c-MET 43
c-myc 1385
Cofluent fibroblast layers 934
Cohort effects 152
Collagen 654
Collagen gene expression 1142
Colon adenocarcinoma cell lines 1285
Colon cancer 232, 237, 294, 600, 1274
Colon tumours 1318
Colorectal cancer 62, 122, 133, 190, 287,
305, 367, 874, 1009, 1116
Colorectal cancer, human 496
Combination regimens 609
Combined modality 1206
Combretastatin A-4 1318
Concurrent chemoradiation in NSCLC 310
Core biopsy 1088
Cost-effectiveness analysis 1243
COX-2 1274
CPT-11 95, 440
CT antigens 1162
Keyword index to Volume 81
1436
British Journal of Cancer (1999) 81(8), 1436-1438
(c) 1999 Cancer Research Campaign
Article no. bjoc.1999.0865
Keyword index 1437
British Journal of Cancer (1999) 81(8), 1436-1439
(c) Cancer Research Campaign 1999
CTLs 342, 881
Cultures 1344
Cyclic A 1017
Cyclic D1 127
Cyclin D 705
Cyclin D1 1174
Cyclin-dependent kinase 705
CYP17 polymorphisms 141
Cystoprostatectomy 832
Cytochrome P450 reductase 1127
Cytogenetics 6
Cytokeratin 8 769
Cytokeratin 20 870
Cytokines 359
Cytokine-induced killer cells 1009
Cytological screening 159
Cytoplasm 1174
Cytoreduction 733
Cytotoxicity 367, 1022, 1127
DCIS 1410
Dendritic cells 1280
Dialysis in vitro 330
Diet 1234, 1238
Differentiation markers 855
Directly streptavidinylated antibodies 431
Dissemination 43
Distinct entities 586
Divalent Fab 972
DNA damage 1127
DNA intercalation 367
DNA mutations 409
DNA repair 800
Docetaxel 457, 609
Docosahexaenoic acid 80, 1238
Dose escalation 1022
Dosimetry 972
Doxorubicin 966
Drug binding 783
Drug delivery systems 1155
Drug targeting 404
Ductal carcinoma in situ 702
Early relapse 1385
E-cadherin 1103, 1392
ED-B fibronectin hybridization in situ 1071
EGF 1134
Eicosapentaenoic acid 80, 1238
Electromagnetic fields 377
ELISA 490
Endotoxins 1311
Ependymoma 1150
Epidemiology 62, 144, 672, 900
Epstein-Barr virus 1182
Establishment 814
Esthesioneuroblastoma 586
Evaluations 912
Experimental metastasis 756
External ear 528
Extracellular matrix 1142
Extracellular signal regulated kinase 1116
Extraction 600
False-negative tests 305
Familial clustering 1150
FAP 232
-Fetoprotein 1188
Fibronectin isoforms 1071
FISH 1165, 1328
Fish oil 80, 440
Flavopiridol 269
Fluorescence 600
Fluorouracil derivatives 484
Free fraction 330
G1 checkpoints 696
Gastric cancer 19, 484, 647, 1356, 1392
G-CSF 1031
Gemcitabine 609, 846
Gene transfer 1009, 1122
Gene-specific repairs 1294
Genetics 672
Genetic markers 1002
Genetic susceptibility 179, 1262
Geographic variations 1262
Germlines 409
GF120918 942
Glioblastoma 994
Glioma 835, 1371
Glucose 1,6-bisphosphate 219
Glucosylceramide 423
Glutathione 75, 796, 989
Glycolysis 219
Glycosylation 1188
Granzyme B 881
Growth inhibition 952
Haemangiosarcomas 532
Haematopoiesis 808
Haemoccult IIR 305
Haemophilus influenzae 175
H2-Antagonists 1356
HBsAg carriers 69
HBV profiles 69
Head and neck cancer 323, 457, 1196
Head and neck tumours 677
Hearts, chicken 934
Hepatocellular carcinoma 393, 747, 814,
1188
Hepatocyte growth factor 721
HER2/neu 1419
Hexokinase 219
HGF 194
HGF/SF 43
hGRPR 966
HHV-8 893
High-dose sequential chemotherapy 449
Histological grades 167
Histology 152
HIT genes 874
HLA 342
hNMBR 966
Hodgkin's disease 476, 1182
Hormone analogues 966
HPV 114
HT-29 294
HT-29 cells 367
Human topoisomerase I 800
Human cultured dendritic cells 1280
Human herpesvirus-8 893
Humanized monoclonal antibodies 1419
Hybrid capture 554
Hybridization in situ 496, 712
17-Hydroxylase/C17,20
-lyase inhibitors 622
Hypermethylation 677
Hypoxia 989, 1127
ICE 277, 1031
I1307K 850
Image analysis 1363
Image cytometry 989
Imbalances 684
Immortalization 377
Immunoassays 122
Immunoconjugates 1155
Immunocytochemistry 404, 1213
Immunohistochemistry 127, 747, 1080
Immunotherapy 1080, 1280
In situ hybridization 496, 712
In vitro dialysis 330
Induction chemotherapy 310
Infection 549
Inflammatory breast cancer 449
Inoperability 1031
Interleukin-1 277
Interleukin-1ra 277
Interleukin-2 822, 1009
Interleukin-2/interleukin-2R pathways 822
Interleukin-6 690
Interleukin-8 647
International studies 463
Interphase cytogenetics 684
Interstitial photodynamic therapies 631
Interval cancer 912
Intestinal side-effects 440
Intraluminal seeding 6
Intramedullary tumours 835
Invasion 654, 774
Ionizing radiation 959
Irinotecan 440
(Iso)flavonoids 269
Japan 1248
JC virus 898
JM216 1294
Kaposi's sarcoma associated herpesvirus 893
K+-ATPase 28
Ki-67 747, 1017
Kinase activities 705
KN62 959
Knock out mice 359
KSHV 893
Laminin isoforms 1071
Leukaemia 549, 833, 898, 1162, 1398
Leukaemia, acute lymphoblastic 175
Leukaemia, acute myeloid 900
Lipopolysaccharides 1311
Liver metastasis 600
LNCaP cells 242, 622
Lobular breast cancer 1103
Locally advanced cancer 1031
Locoregional recurrence 1385
LOH 108, 503, 677, 1103, 1111, 1371, 1410
Loss of heterozygosity 108, 503, 677, 1103,
1111, 1371, 1410
Luminometric immunoassays 122
Lung cancer 705, 1127, 1243
Lung surgery 721
Lymphangiogenesis 54
Lymphangiosarcomas 532
Lymphocyte function 1182
Lymphoedemas 532
Lymphomas 1009
B-Lymphoma cells, human 43
Lysosomes 367
Macrophages 37, 496
MACS 1165
1438 Keyword index
British Journal of Cancer (1999) 81(8), 1436-1439 (c) Cancer Research Campaign 1999
Magnetic resonance imaging 616, 638
Male breast cancer 141
Malignant mesothelioma 54, 1111, 1344
Mammalian cells 377
Mammary cancer 409, 1335
Mammary tumours 194
Mammographic parenchymal patterns 1257
MAPK 1134
Marimastat 19
Markers 741
Mastectomy 1222
Matrigel 934
Matrix metalloproteinases (MMPs) 19, 287,
774
MCP-1 855
MEK 1116
Melanomas 219, 774, 918, 1066
Meningioma 381
Menopause 225
MES-Dx5 cells 942
[131I]-Metaiodobenzylguanidine 1378
Metallothionein 712
Metastasis 287, 316, 712, 774, 814, 832,
1274, 1311
Metastasis-associated factors 814
Metastatic potential 756
Methotrexate 316, 1037
MHC class I 881
MHC class II 808
MIA 1066
2
-Microglobulin 881
Micrometastases 870, 1002, 1213
Microsatellites 1371
Microsatellite instability 108, 190
Microsatellite marker analysis 1111
Microvessels 484
Microvessel count 1088
Microvessel density 1363
Minimally invasive debulking 631
Mismatched DNA 212
Mitoxantrone 316
MMP 1274
MN/CA IX/G250 antigens 741
MN/CA9/G250 genes 741
Models 1243
Molecular analysis 586
Molecular cytogenetics 1328
Monoclonal antibodies 1155
Monoclonality 6
MRP1 269
MSI 108, 190
mTHPC 37
MTS 1280
MUC1 mucin 431
Multidrug resistance 269, 423, 783, 942,
1304
Multifocal uroepithelial carcinomas 6
Multiple myeloma 952
Murine tumours 1311
Mutations 237, 1103
Myelodysplasia 1398
Myeloma 1162
Myofibroblasts 1071
NADPH 1127
Nasopharyngeal carcinoma 1122
Neoadjuvant chemotherapy 95, 1206
Neoplasms 684
Neoplasms, breast 907
Neoplasm staging 167
Nephrotoxicity 336
Neuroblastoma 1378, 1402
Neuroendocrine carcinoma 1351
Neurotic disorders 907
NFB 981
Nipple aspirate fluid 1222
Nitric oxide 37
NK cells 881
nm23 469
Node-negative breast carcinoma 727
Non-Hodgkin's lymphoma 144, 860
Non-seminomatous germ cell tumours 1188
Non-small-cell lung cancer 127, 510, 769,
1031
Non-small-cell lung cancer, advanced 846
Non-small-cell lung cancer cell lines 609
Non-small-cell lung cancer primary cultures
609
Non-specified abuse 907
Non-steroidal anti-inflammatory drugs 62,
542
NSAIDs 62, 542
Nucleus 1174
Nude rats 1344
Nutritional supplementation 80
Obesity 890
Oesophageal cancer 712
Oesophageal squamous cell carcinoma 469
17-Oestradiol 387
Oestrogen 918
Oestrogen receptors 1042
Oestrogen replacement therapy 559
Oncofetal extracellular matrix 1071
Oncogene amplification 1328
Oral, gastric and colon cancer cells 416
Oral carcinoma 881, 1022
Oral contraceptives 918
Oral squamous cell carcinoma 1071
Orthotopic transplantation 934
Orthotopic tumours 1318
Ovarian cancer 152, 179, 631, 662, 733, 790,
855, 1174, 1304
Ovarian neoplasms 559, 654
Overall surival 1385
Overweight 890
P4 952
p16 677, 1122
p21 133
p21WAF1 959
p21wafl 252
p27Kip1 gene 1052
p53 179
p53 122, 133, 490, 702, 733, 959, 994, 1285
p53, canine 409
p53 mutants 212
Paclitaxel 416
Paediatric cancer 300
PAK-200S 1304
Pancreas-specific genes 350
Pancreatic cancer 80, 350
Papillomaviruses, human 554, 1234
PEG-m-THPC 631
Peptide 342
Perforin 359
Pericarditis 1037
Period effects 152
Peripheral blood 350
Peritonitis 1037
P-glycoprotein 783
P-glycoprotein inhibitors 942
pgp-170 416
Phagocytosis 37
Pharmacokinetic-pharmacodynamic
relationships 330
Pharmacokinetics 261, 1022, 1419
Phase I studies 1419
Phase I trials 760
Phase II studies 310
Phase II trials 457
Phenacetin 542
Phenotypic markers 952
Phosphofructokinase 219
Photodynamics 37
Photodynamic therapies 261, 520, 600, 616
Photosensitizers 261, 600
Phthalocyanines 616
Physiological factors 925
Phyto-oestrogens 1248
PK1-doxorubicin 99
Plasma ultrafiltrate 330
Plasminogens 1269
Platinum-based therapies 75
Pleural effusions 1059
Pleurisy 1037
PML 994
Pneumonitis 1037
Polymer conjugates 261
Polymorphism 179, 850
Population mixing 144
Population pharmacokinetics 99
Population-based screening 912
pPNETs-ETs 586
PR-39 393
Preclinical therapies 609
Preclinical toxicology 760
Predictive assays 354
Premenopause 167, 918
Prevention 1243
Primary chemotherapy 841
Prodrug therapy 24
Prognosis 122, 127, 133, 190, 469, 510, 662,
855, 1052, 1182, 1213
Prognostic factors 667, 747
Programme sensitivity 305
Progression 127
Prolactin-inducible proteins 1002
Proliferation 1017
Proliferative tumour cells 925
Proportionate incidences 912
Prostaglandin E2
194
Prostate cancer 28, 242, 1052, 1269
Prostate cancer, androgen-dependent 28
Prostate cancer, androgen-independent 28
Prostate-specific antigens 490, 1269
Prostatic neoplasms 672, 1238
Proteases 790
Proteins 490
Protein kinase A 1134
Protein structures 1188
Proteolysis 287
Proteolytic cleavage 212
Proto-oncogenes 237
Proxy respondents 87
Purification 1188
17q24 1402
Q-PCR-EIA 114
Quality of life 87
Keyword index 1439
British Journal of Cancer (1999) 81(8), 1436-1439
(c) Cancer Research Campaign 1999
Questionnaires 87
Quiescent tumour cells 925
RAD51 503
RAD52 503, 800
RAD54 503
Radiation 532
Radioimmunotherapy 972
Radiometabolic therapies 1378
Radiosensitivity 959
Radiotherapy 108, 323, 727, 841, 1196
Radon 900
Radon remediation 1243
Raf-1 1116
Randomization 1356
Ras 237
Ras 1116
Rat 631
RB phosphorylation 808
Rb protein 342, 705
RBP-1 342
Rectal neoplasms 463
Registries 463
Relapse 727
Relapse-free survival 1385
Renal cancer 1009
Renal cell cancer 542, 741
Renal function 336
Residual diseases 860
Resistance 252
Response to chemotherapy 733
Retinoic acid 381
Retrospective cohort studies 144, 1351
Retroviral transduction 43
Reverse transcription polymerase chain
reaction 350, 741, 870, 966, 1002, 1066
RT-PCR 350, 741, 870, 966, 1002, 1066
S phase 404
S phase fractions 1017
sCD30 1182
Schedules 1285
SCID mice 43, 622
Screening 305
Seascale 144
Seasonality 549
Second-line chemotherapy 846
Secondary myeloid malignancy 476
Selective COX-2 inhibitors 1274
Seroprevalence 893
Serosal complications 1037
SF2 354
s-ICAM-1 1182
Sigma-2 receptors 925
Single-chain antibodies 790
Skin 528
Small-cell lung cancer 667, 1206, 1213
SN-38 1285
Sodium 28
Soft-tissue sarcomas 890, 1017
Solid tumours 99
Soluble receptors 203
Somatostatin receptor 2 1402
South-east Asia 893
Southern blot analysis 1410
Soya 1248
Spinal cord tumours 835
Squamous cell carcinoma 95, 159, 510, 528
SSTR2 gene 1402
Stage III cancer 122
Staging 463
Standardization 870
Starting doses 760
Steroids 690
Steroid hormones 225, 783
Stewart-Treves syndrome 532
Superantigens 359
Surgery 323, 1206, 1311
Survival 323, 528, 727, 850
Survival analysis 463
Symptoms 1196
Syndecan 393
t(14;18) 860
Targeting therapy 1155
T-cells 359
Testicular cancer incidence 1262
Thymidine phosphorylase 484
Thymomas 841
Tirapazamine 1127
Tissue inhibitor of matrix metalloproteinase
(TIMP) 774
TNF- 37
Tobacco 1228
Tomudex 252
Topical application 13
Topoisomerase I 800
Topotecan 1304
Transcription 874
Transfection 647
Transitional cell carcinoma 638
Transplantation 476, 1344
Transurethral resection 832
Treatments 1351
Trivalent Fab 972
Tumorigenesis 232
Tumorigenicity 756
Tumours 874
Tumours, human 75
Tumour antigens 19
Tumour cell growth 822
Tumour cell shedding 756
Tumour immunotherapy 431
Tumour inhibition 966
Tumour invasion 194, 381
Tumour markers 667, 1059
Tumour perfusion 756
Tumour progression 242, 496
Tumour-rejection antigens 1080
Tumour responses 600
Tumour suppressor genes 1103, 1122, 1150
Tumour targeting 966, 972
Tumour therapies 359
Tumour vascular endothelium 1155
Tumour vasculature 261
Tumour-stromal interaction 194
Ultrasound imaging 520
United Kingdom 1243
uPAR 203
Urbanization 1262
Urinary bladder 1363
Vaccination 175
Vascular endothelial growth factor 54, 225,
727, 1335, 1398
VEGF 54, 225, 727, 1335, 1398
VEGF receptors 1335
VEGF-C 54
Weight gain 167
Western blotting 1080
wnt genes 496
Wortmannin 959
Xenografts 774
Yeast 800

				

